# Multiresistant *Neisseria gonorrhoeae*: a new threat in second decade of the XXI century

**DOI:** 10.1007/s00430-019-00651-4

**Published:** 2019-12-04

**Authors:** Beata Młynarczyk-Bonikowska, Anna Majewska, Magdalena Malejczyk, Grażyna Młynarczyk, Sławomir Majewski

**Affiliations:** 1 Department of Diagnostics of Sexually Transmitted Diseases, Medical University of Warsaw, 82a Koszykowa Str, 02-008 Warsaw, Poland; 2 Department of Medical Microbiology, Medical University of Warsaw, 5 Chalubinskiego Str, 02-004 Warsaw, Poland; 3 Department of Dermatology and Venereology, Medical University of Warsaw, 82a Koszykowa Str, 02-008 Warsaw, Poland

**Keywords:** Epidemic clones NG-MAST, Gonorrhoea, Mechanisms of antibiotic resistance, Multiresistant *Neisseria gonorrhoeae*

## Abstract

*Neisseria gonorrhoeae* is an etiologic agent of gonorrhoea, one of the most common sexually transmitted diseases caused by bacteria. For many years, infections caused by *N. gonorrhoeae* were considered to be relatively easy to treat; however, resistance has emerged successively to all therapeutic agents used in treatment of the disease, e.g., penicillin, ciprofloxacin or azithromycin. Currently, the global problem is the emergence and a threat of spread of *N. gonorrhoeae* strains resistant to extended-spectrum cephalosporins (ESC), such as injectable ceftriaxone and oral-used cefixime. Especially, dangerous are multi-resistant strains resistant simultaneously to ESC and azithromycin. Three strains with high-level resistance to azithromycin and resistant to ESC were first time isolated in 2018. Moreover, in 2018, the first ESBL was described in *N. gonorrhoeae* and that makes the threat of appearing the ESBL mechanism of resistance in *N. gonorrhoeae* more real, even though the strain was sensitive to ceftriaxone. Molecular typing revealed that variants resistant to ESC occurred also among strains belonging to epidemic clonal complex CC1 (genogroup G1407) distinguished in NG-MAST typing system. The G1407 genogroup, in particular the ST1407 sequence type, is currently dominant in most European countries. The presence of different mechanisms of drug resistance significantly affects clinical practice and force changes in treatment regimens and introduction of new drugs.

## Introduction

Gonorrhoea is one of the most common sexually transmitted bacterial diseases in the world [1]. Several bacterial or viral diseases can be transmitted simultaneously that may need the adequate diagnostics and therapy [2, 3]. Gonorrhoea diagnostics can be carried out using both culture and molecular methods, but bacterial culture is necessary to determine drug susceptibility [4, 5]. According to the WHO data, the number of gonorrhoea patients amounted to over 78 million people per year [6], and this value may be greatly underestimated considering a relative ease of treatment and incomplete registration of infections in many countries. Single doses of third-generation cephalosporins, such as ceftriaxone or cefixime are used in the treatment of gonorrhoea in the majority of countries in the world. The emergence of *N. gonorrhoeae* strains resistant to ceftriaxone and cefixime, and additionally resistant to penicillin, fluoroquinolones, tetracyclines and azithromycin, on different continents in 2007–2018 aroused great concern around the world [7–10]. This was the reason for the search for new drugs or alternative therapies active against *N. gonorrhoeae.* An example of new inhibitors of bacterial topoisomerase, that could be used in the treatment of gonorrhoea are fluoroquinolones: delafloxacin, sitafloxacin or two drugs in clinical investigations, zoliflodacin and gepotidacin. Examples of protein synthesis inhibitors are new drugs that entered clinical trials: lefamulin and solithromycin and drugs only in preclinical tests: apramycin (aminoglycoside) and aminomethyl spectinomycins [11–18]. Another candidate for gonorrhoea treatment with a new mechanism of action, a small antimicrobial molecule SMT-571, demonstrated in vitro high activity against all investigated strains of *N. gonorrhoeae* [19]. Aside from usage of old and new drugs alone or in combinations, alternative therapies with probiotics or bacteriophages are taken into consideration. Antigenic variability makes the development of gonococcal vaccine difficult. Only a few vaccines entered clinical trials and all of them were unsuccessful. Recently, the animal trials with LOS-derived OS epitope (2C7) gave the promising results. Another interesting fact is that persons vaccinated against serogroup B meningococci are partially immune against gonorrhoea [20, 21] In 2017, gonococci were included into the WHO list of 12 pathogens, whose drug resistance is a global health threat, and which most urgently require the creation of new antibiotics [22].

## Spreading of multi-drug resistant variants of *N. gonorrhoeae* and their importance

The problem of bacterial drug resistance can be considered at various levels. It is particularly important to understand that the evolution of resistance mechanisms in bacteria is inextricably associated with the epidemiology of infections with a given species. The emergence of a new resistance mechanism is much more important if it occurs in specific epidemic or pandemic clones, which for various reasons are characterized by increased infectiveness and/or pathogenicity and are, therefore, responsible for a majority of infections with a particular species. The representatives of the CC1 clonal complex NG-MAST (G1407 genogroup NG-MAST) [23] according to the molecular typing system NG-MAST (*Neisseria gonorrhoeae* multi-antigen sequence typing), which is most frequently used in the epidemiological studies of *N. gonorrhoeae* (MLST, NG-STAR, MLVA and other systems are also used), seem to play the dominant role in *N. gonorrhoeae*. The G1407 genogroup, in particular the ST1407 sequence type, is currently dominant in most European countries. This genogroup includes about 330 sequence types, also including ST3128, ST3158, ST4120, ST5332, which dominate or have a significant share in individual countries [24, 25]. The interest in resistance mechanisms remains closely related to clinical practice. The emerging resistance mechanisms force the introduction of new drugs and change of treatment regimens. The timetable of appearance of the resistance in *N. gonorrhoeae* to particular drugs is shown in the Table 1 [7, 15, 26–30]. The arising, presently still rare in this species, resistance to third-generation cephalosporins is of particular importance for *N. gonorrhoeae*. However, it was observed that the mechanisms of resistance to drugs formerly used in gonorrhoea treatment, i.e., plasmid-coded, once very popular (such as the resistance to penicillin related to beta-lactamase production or TetM proteins related to tetracycline resistance) do not occur at all or are very rare in the currently dominating sequence types in Europe. In these bacteria, mechanisms involving the synthesis of specific plasmid-coded proteins have been replaced by other mechanisms, conditioned by mutations of chromosomal genes. The transfer of beta-lactamase plasmid to *N. gonorrhoeae* belonging to genogroup ST1407 is possible, but the bacteria cannot maintain the plasmid for longer time [31]. This indicates a tendency of removing the energy-intensive ballast by bacteria in the absence of selective pressure in the environment. The level of resistance to a specific antibiotic, measured by the MIC value, is much lower in these cases. For this reason, it is especially important to adopt appropriate criteria for recognizing a bacterial strain as resistant or susceptible to a particular drug. Generally accepted criteria are determined annually by institutions appointed for this purpose, such as EUCAST (The European Committee on Antimicrobial Susceptibility Testing), obligatory in many European countries, and CLSI (Clinical and Laboratory Standards Institute), obligatory in the USA and many non-European countries. These criteria may differ from each other and may change in successive years. In European countries, accuracy of antimicrobial susceptibility tests is checked by appropriate and regular quality assurance procedures [32]. Current EUCAST and CLSI (March 2019) breakpoints are presented in Table 2 [33, 34]. Therefore, when specifying the percentage of bacteria susceptible to a given antibiotic, it is necessary to provide the accepted susceptibility criteria, or to calculate the MIC_50_ and MIC_90_ values. The term “resistance” is abused in relation to certain mechanisms. In *N. gonorrhoeae*, for example, the mechanism related to the overproduction of MtrCDE, MacAB or NorM membrane pumps, unless it interacts with another mechanism, does not cause resistance, but only decreases the susceptibility (increase in the MIC value of the drug) and the strain is classified as susceptible.

**Table 1:** The year of introduction of drug and first report of resistance

Drug	Introduction (year)	Resistance	
		Year and country	Mechanism
Sulfonamides	1930	1945	Probably chromosomal mutation
Penicillin	1943	1945 1980 Canada 1976 England, USA	Probably chromosomal mutation Chromosomal mutation Plasmid encoding beta-lactamase (PPNG strains)
Tetracyclines	1949	1969 USA 1985 USA	Probably chromosomal mutation Plasmid encoding TetM
Spectinomycin	1967	1973 England 1981 England	Chromosomal mutation Spectinomycin resistant PPNG
Ciprofloxacin	1980	1990 USA	Chromosomal mutation
Azithromycin	1988	1991 USA 1997 USA	*ermF* Chromosomal mutation 23S rRNA or *mtr*
CME Ceftriaxone Cefixime	1989	2007 China 2009 Japan 2010 France 2018 England Australia	Chromosomal mutation *penA* (ceftriaxone MIC = 0.5 mg/L) Chromosomal mutation *penA* (ceftriaxone MIC = 2.0 mg/L) Chromosomal mutation *penA* (ceftriaxone MIC = 1.0 mg/L) Chromosomal mutation *penA* and 23S rRNA (ceftriaxone MIC = 0.5 mg/L and azithromycin MIC > 256 mg/L)

**Table 2:** MIC breakpoints for *Neisseria gonorrhoeae*

Drug	MIC breakpoints (mg/L)			
	EUCAST 2019		CLSI 2019	
	*S* ≤	*R* >	*S* ≤	*R* ≥
Benzylpenicillin	0.06	1.0	0.06	2.0
Cefixime	0.125	0.125	0.25	–
Cefotaxime	0.125	0.125	0.25	–
Ceftriaxone	0.125	0.125	0.25	–
Ciprofloxacin	0.03	0.06	0.06	1.0
Ofloxacin	0.125	0.25	0.25	2
Azithromycin*	1.0	1.0	1.0	–
Tetracycline	0.5	1.0	0.25	2.0
Spectinomycin	64	64	32.0	128.0

*Azithromycin is always used in conjunction with another effective agent

## Acquisition of drug resistance by *N. gonorrhoeae*

The mechanism of acquiring resistance is another area, in which the problem of resistance to antibacterial drugs can be considered. The acquisition of a new mechanism of resistance by epidemic clones in the majority of both Gram-positive and Gram-negative bacteria occurs as a result of external uptake, usually by conjugation, of mobile genomic elements (plasmid, transposon, gene cassettes integrated into integron, genomic resistance islands, etc.). The transmission of chromosomal genes with mutations that occurred in the donor through transformation in natural conditions has been described only in the genus *Neisseria* and to a much smaller extent in *Streptococcus*. Resistance or reduced susceptibility to antibacterial drugs in currently dominant *N. gonorrhoeae* clones is most often associated with mutational changes in chromosomal genes. One or more mutations (so-called non-mosaic changes involving one or several amino acids in the PBP2 protein) or a combination of multiple mutations, the effect of which is a change from 21 to more than 60 amino acids in the coded protein (so-called mosaic or semimosaic changes), are required for the emergence of resistance in some genes (e.g., *penA*). The unique ability among bacteria of the genus *Neisseria* to uptake chromosomal DNA with a high frequency through transformation from other genera of *Neisseria* allows to accumulate many mutations in chromosomal genes. Most bacteria need to acquire the state of competence to uptake DNA through transformation. Bacteria from the genus *Neisseria* remain constantly in the state of competence, and moreover, *Neisseria* chromosomal DNA has specific sequences, known as 10 bp DUS (DNA uptake sequence), and GCCGTCTGAA sequences that act as a generic-specific DNA marker. There are 1965 copies of the DUS sequence in a 2.15-Mb chromosome, which means that they occur on average every 1096 bp [35]. DUS sequences enable bacteria, which uptake DNA by means of transformation, to determine if it is derived from the genus *Neisseria*. Extrachromosomal DNA (plasmids) does not contain DUS markers and is transformed at a much lower frequency. Plasmid transmission is most often carried out by conjugation (conjugative plasmids, non-conjugative plasmids mobilized by conjugative elements). The most important is the uptake of altered genes or their fragments, determining conditioning resistance to drugs, through *N. gonorrhoeae* or *N. meningitidis* epidemic or pandemic clones. Not every mutation that reduces antibiotic susceptibility, taken up through transformation, even by an epidemic clone, guarantees evolutionary success. It should be noted that the mutation, despite the fact that it determines resistance (which guarantees the clone an advantage in the case of selective pressure, e.g., during treatment), may also have a negative impact on the physiological functions of the altered protein and related proteins, e.g., within various regulatory systems. Reduced or altered metabolic activity of some proteins may affect infectivity, colonization capacity, metabolic rate, mechanisms that protect the cell against the immune system (quantitatively or qualitatively), which may be a significant obstacle in gaining a quantitative advantage in confrontation with clones without metabolic defects.

## Resistance to beta-lactams

Beta-lactam antibiotics have a bactericidal effect against *N. gonorrhoeae*, as a result of inactivation of the transpeptidase domain in PBP2 and PBP1 proteins involved in peptidoglycan synthesis. Penicillin action is based on acylation of serine at position 310 in *N. gonorrhoeae* PBP2 [36]. The penicillin MIC for strains with chromosomal resistance to penicillin is most often between 2.0 and 4.0 mg/L, and from 4.0 to 32.0 mg/L for beta-lactamase-producing strains. Strains with MIC ≥ 0.25 mg/L are considered resistant to ceftriaxone and cefixime according to the European criteria. CLSI did not specify criteria for resistant strains (Table 2).

## Chromosomal resistance to penicillin and oxyimino-cephalosporins

The first *N. gonorrhoeae* strain with chromosomal resistance to penicillin was described in the USA in North Carolina in 1983 [37]. Currently, the global problem is the threat of emergence and spread of *N. gonorrhoeae* strains resistant to extended-spectrum cephalosporins (ESC), such as ceftriaxone and cefixime. However, only a few strains with ESC resistance have been described so far. The first such strain, HO41 resistant to cefixime (MIC = 4 mg/L) and ceftriaxone (MIC = 2 mg/L), was isolated in 2009, in Kyoto, Japan [29, 38]. No further ESC-resistant *N. gonorrhoeae* strains were found in the Kyoto and Osaka regions in 2010–2012, indicating that HO41 was not an epidemic clone and did not spread in the population [39]. However, some other resistant strains appeared in different countries. The *N. gonorrhoeae* strains with ceftriaxone MIC ≥ 0.5 mg/L are presented in Table 3 [7, 8, 29, 30, 40–51].

**Table 3:** *N. gonorrhoeae* strains with ceftriaxone MIC ≥ 0.5 mg/L

Country	Year	Strain	ST NG-MAST	Genogroup NG-MAST	ST MLST	ST NG-STAR	PenA	CTX MIC mg/L
Ireland	2018	IR72	17,842	G564	1903	1133	60.001	0.5
Australia	2018	A2543*	16,848	G1866	12,039	996	64.001	0.5
England	2018	G97687*	16,848	G1866	12,039	996	64.001	0.5
England	2018	G7944*	16,848	G1866	12,039	996	64.001	0.5
France	2017	F90	3435	G564	1903	133	60.001	0.5
Denmark	2017	GK124	1614	G5267	1903	ND	ND	0.5
Canada	2017	47,707	1614	G5267	1903	233	60.001	1.0
Australia	2017	A7536	15,925	G11110	1903	233	60.001	0.5
Australia	2017	A7846	1614	G5267	1903	233	60.001	0.5
Japan	2015	FC428	3435	G564	1903	233	60.001	0.5
Japan	2015	FC460	3435	G564	1903	233	60.001	0.5
Japan	2014	GU140106	6543	G11018	7363	ND	ND	0.5
Argentina	2014	CCETS-7069	13,064	G21	13,637	139	IX	0.5
Australia	2013	A8806; WHO-Z	4015	G11018	7363	227	64.001	0.5
China	2012/13	GD4	10,208	G5062	ND	ND	II	0.5
China	2012/13	GX1	11,129	ND	ND	ND	XXI	0.5
China	2012/13	HN9	5913	G1791	ND	ND	XXI	0.5
Spain	2012	F89; WHO-Y	1407	G1407	1901	16	42.001	1.0
France	2010	F89; WHO-Y	1407	G1407	1901	16	42.001	1.0
Japan	2009	HO41;WHO-X	4220	G4019	7363	226	37.001	2.0
China	2007	ND	2288	G1791	ND	ND	XVII	0.5

*ND* not done, *CTX* ceftriaxone

*Azithromycin resistance, MIC > 256 mg/L

In 2018, three strains resistant to ceftriaxone and azithromycin (MIC = 256 mg/L) were described in Great Britain and Australia (two strains) [44, 46, 52]. In 2011–2015, 26 strains resistant to ceftriaxone (MIC > 0.125 mg/L) were detected as part of the Euro-Gasp program. These strains occurred in Germany (*n* = 10), Spain (*n* = 6), Greece (*n* = 4), Austria (*n* = 3) and one strain in Norway, Ireland and Slovenia [53]. Fifteen strains resistant to ceftriaxone were described as a result of retrospective studies of strains isolated in China in 2007 [7]. Subsequent studies of *N. gonorrhoeae* strains allowed characterization of another 29 strains resistant to ceftriaxone [8].

The reported increased in *N. gonorrhoeae* MICs of the ESC currently used in gonorrhoea treatment, ceftriaxone and cefixime and of penicillin are probably the effect of mutations in the chromosomal region of the *penA* gene (encoding the transpeptidase domain of the PBP2 protein), *porB1b* gene (encoding the porin B subunit), *ponA* gene (encoding the PBP1 protein) and overexpression of MtrCDE membrane pump proteins. Mutational changes in the *penA* gene play the most important role in the emergence of chromosomal resistance or reduced susceptibility to ESC. The mutated *penA* gene is probably acquired by transformation from commensal bacteria *Neisseria cinerea* [54]. The resulting changes in the amino acid pattern of the PBP2 protein (most often substitutions and single deletions and insertions of amino acids) are referred to as PBP2 mosaic, semimosaic or non-mosaic patterns. Sixty-four patterns of the PBP2 protein are described (85–576 amino acid region). Amino acid sequences of the PB2-2A protein of the *N. gonorrhoeae* M32091 strain were assumed as the wild type (pattern 0). Patterns I–IX, XI–XXII, XXIV, XXXIII, 40, 41, 43–46, 48–50, 54, 56, 57 and 61, are defined as non-mosaic (number of amino acid changes from 1 to 13). Patterns XXIII, XXXV and 39 were defined as semimosaic (number of amino acid changes from 22 to 31). The remaining ones are considered mosaic patterns (number of amino acid changes from 33 to 61) [23]. The described PBP2 patterns determine different MIC values of ESC and penicillin [23, 55, 56]. The increase in ESC MIC (reduced susceptibility or resistance to ESC, ceftriaxone MIC of 0.125–0.25 mg/L) is characteristic for some *N. gonorrhoeae* carrying PBP2 not only with changes, referred to as mosaic patterns (amino acid substitutions probably conditioning resistance or reduced susceptibility are: A311V, I312M, V316T or P, T484S, A502P, G546S) occurring more frequently in European countries, but also with non-mosaic patterns containing substitutions: A502V, A502T, G543S, P552S in PBP2 and usually a D346 insertion (more often in Asian countries) [5, 7, 8, 57, 58]. The HO41 and F89 strains, showing high resistance to ESC, have the PBP2 37 (former name: C) and 42 mosaic patterns (former name: CI). PBP2 patterns, according to Demczuk et al. [23], including amino acid substitutions and insertions determining reduced susceptibility or resistance to ESC are presented in Table 4 [7, 8, 23, 29, 30, 47, 48].

**Table 4:** Changes of the amino acids in the *N*.*gonorrhoeae* PBP2 protein transpeptidase domain including sites determining reduced sensitivity or resistance to oxyimino-cephalosporins

No PBP2	NC/TC	Selected changes (aa)									No PBP2	NC/TC	Selected changes (aa)								
		A	I	V	□	T	A	G	G	P			A	I	V	□	T	A	G	G	P
		3	3	3	3	4	5	5	5	5			3	3	3	3	4	5	5	5	5
		1	1	1	4	8	0	4	4	5			1	1	1	4	8	0	4	4	5
		1	2	6	6	4	2	3	6	2			1	2	6	6	4	2	3	6	2
I	1/n	▄	▄	▄	D	▄	▄	▄	▄	▄	XXXIII	7/n	▄	▄	▄	D	▄	V	S	▄	▄
II** II_+502V_**	5/n 6/n	▄	▄	▄	D D	▄	▄ V	▄	▄	▄	XXXIV** +552S +502V	52/m 53/m 53/m	▄	M M M	T T T	▄	▄	▄ V	▄	S S S	▄ S
III	4/n	▄	▄	▄	D	▄	V	▄	▄	▄	XXXV	31/sm	▄	M	T	▄	▄	▄	▄	▄	▄
IV	5/n	▄	▄	▄	D	▄	▄	S	▄	▄	XXXVI*	33/m	▄	▄	▄	▄	▄	▄	▄	S	▄
V*	6/n	▄	▄	▄	D	▄	▄	S	▄	▄	37**	61/m	▄	▄	▄	▄	▄	▄	▄	▄	▄
VI	4/n	▄	▄	▄	D	▄	▄	▄	▄	L	38	33/m	V	M	T	▄	S	▄	▄	▄	▄
VII	5/n	▄	▄	▄	D	▄	V	S	▄	▄	39	26/sm	▄	▄	▄	D	▄	▄	▄	▄	▄
VIII	4/n	▄	▄	▄	D	▄	V	S	▄	L	40	2/n	▄	▄	▄	D	▄	▄	▄	▄	▄
IX**	4/n	▄	▄	▄	D	▄	▄	▄	▄	L	41	13/n	▄	▄	▄	D	▄	▄	S	▄	▄
X	59/m	▄	M	T	▄	▄	▄	▄	S	▄	42**	53/m	▄	M	T	▄	▄	P	▄	S	▄
XI	6/n	▄	▄	▄	D	▄	V	▄	▄	L	43	5/n	▄	▄	▄	D	▄	V	▄	▄	▄
XII*	5/n	▄	▄	▄	D	▄	▄	▄	▄	S	44	6/n	▄	▄	▄	D	▄	T	▄	▄	L
XIII*	6/n	▄	▄	▄	D	▄	V	▄	▄	S	45	10/n	▄	▄	▄	▄	▄	▄	▄	▄	▄
XIV	5/n	▄	▄	▄	D	▄	▄	▄	▄	▄	46	5/n	▄	▄	▄	D	▄	▄	▄	▄	▄
XV	1/n	▄	▄	▄	▄	▄	▄	▄	▄	▄	47	39/m	▄	M	T	▄	▄	▄	▄	▄	▄
XVI	6/n	▄	▄	▄	D	▄	V	▄	▄	▄	48	11/n	▄	▄	▄	D	▄	▄	▄	▄	▄
XVII**	9/n	▄	▄	▄	D	▄	V	S	▄	▄	49	6/n	▄	▄	▄	D	▄	T	▄	▄	▄
XVIII*	8/n	▄	▄	▄	D	▄	T	S	▄	▄	50	5/n	▄	▄	▄	D	▄	▄	▄	▄	A
XIX	8/n	▄	▄	▄	D	▄	▄	▄	▄	▄	51	55/m	▄	M	T	▄	▄	▄	▄	S	▄
XX	7/n	▄	▄	▄	D	▄	▄	▄	▄	▄	52	54/m	▄	M	T	▄	▄	▄	▄	S	▄
XXI**	12/n	▄	▄	▄	D	▄	V	▄	▄	▄	53	53/m	▄	M	T	▄	▄	▄	▄	S	A
XXII	11/n	▄	▄	▄	D	▄	▄	▄	▄	▄	54	6/n	▄	▄	▄	D	▄	V	▄	▄	A
XXIII	22/sm	▄	▄	▄	D	▄	▄	▄	▄	▄	55	53/m	▄	M	T	▄	▄	▄	▄	S	▄
XXIV	9/n	▄	▄	▄	D	▄	▄	S	▄	S	56	10/n	▄	▄	▄	D	▄	V	▄	▄	G
XXV	58/m	▄	M	T	▄	▄	▄	▄	S	▄	57	7/n	▄	▄	▄	D	▄	V	▄	▄	A
XXVI	52/m	▄	M	T	▄	▄	V	▄	▄	▄	58	54/m	▄	M	T	▄	▄	▄	▄	S	A
XXVII	58/m	▄	M	T	▄	▄	▄	▄	S	▄	59	57/m	V	M	T	▄	S	▄	▄	S	▄
XXVIII	60/m	▄	M	T	▄	▄	▄	▄	S	▄	60**	49/m	V	M	T	▄	S	▄	▄	S	▄
XXIX	58/m	▄	M	T	▄	▄	▄	▄	S	▄	61	6/n	▄	▄	▄	D	▄	▄	▄	▄	L
XXX	59/m	▄	M	T	▄	▄	V	▄	S	▄	62	26/sm	▄	▄	▄	▄	▄	▄	▄	▄	▄
XXXI	60/m	▄	M	T	▄	▄	▄	▄	S	▄	63	44/m	▄	M	T	▄	▄	▄	▄	▄	▄
XXXII	53/m	▄	M	T	▄	▄	▄	▄	S	L	64**	57/m	V	M	T	▄	S	▄	▄	S	▄

Changes aa: ins346, T484, A502, G543, G546, P552 were named in some former works: ins 345a, T483, A501, G542, G545, P551, respectively

(filled square box) insertion-wild type (no change), *NC* number of changes, *TC* type of changes, *m* mosaic, *n* non-mosaic, *sm* semi-mosaic, *aa* amino acids, *A* alanine, *D* aspartic acid, *G* glycine, *I* isoleucine, *L* leucine, *M* methionine, *P* proline, *S* serine; *V* Valine

*Strains with MIC of ceftriaxone = 0.25 mg/L were described; **strains with MIC of ceftriaxone ≥ 0.5 mg/L were described

In addition to mutations in the PBP2 encoding genes, the increase in penicillin MIC may also be conditioned by mutations in the *penC* gene (alternative gene name: *pilQ*), encoding PilQ secretin [59]. However, analyses of strains isolated in South Korea [60] showed that *N. gonorrhoeae* strains exhibiting the same changes in the genes coding for PBP2, MtrR, PBP1 and porin B differed significantly in the MIC of ceftriaxone, cefixime, cefpodoxime and penicillin G. This indicates that these gene alterations do not fully explain the reasons for beta-lactam MICs.

The highest percentage of cefixime-resistant *N. gonorrhoeae* among European countries in 2016 was recorded in Croatia (11.1%), Luxembourg (10%), Belgium (8.1%), Germany (6.4%), Poland (5.2%), Austria (4.2%) and Slovakia (3.6%) [61]. In some European countries, i.e., in Poland, the strains were isolated first time in 2016; while, an increase in the number of strains with reduced susceptibility to ceftriaxone (MIC = 0.125 mg/L) and decrease in penicillin-susceptible strains were observed in previous years [62, 63]. No cases of resistance to ceftriaxone were detected in 2016 in Europe [61].

## Plasmid mediated resistance to penicillins

The first *N. gonorrhoeae* producing beta-lactamase was described in Great Britain in 1976 [64]. Since then, until 2018, nine different beta-lactamases were isolated from *N. gonorrhoeae*, all from the TEM group. Beta-lactamases are encoded by a family of non-conjugative penicillin plasmids that differ in deletions, insertions or duplications. These changes are related to the described earlier Asia plasmid (4.4 MDa, 7426 bp, pJD4 prototype). Plasmids described so far include: Africa (3.2 MDa, 5599 bp, 1880–3708 deletion, pJD5 prototype), Toronto/Rio (3.05 MDa, 5154 bp, 3802–6075 deletion, pJD7 and pGO4717 prototypes), Nimes (3.8 MDa, 6798 bp, 1880–3708 deletion and IS5 insertion 1200 bp between nucleotides 604–605, pGF1 prototype), New Zealand (8.5 MDa, 9309 bp, TR duplication tandem repeat, 1883 bp from nucleotide 593), Johannesburg (4865 bp, two deletions: 1928–4487 and 6236, pEM1 prototype), Australia (3269 bp, two deletions: 1928–4487 and 3795–6066 and T7424C transitions) [65–69].

Beta-lactamases described in *N. gonorrhoeae* are: TEM-1, occurring in all types of penicillinase plasmids previously described in *N. gonorrhoeae*, TEM-135 (M182T substitution) most commonly found in the Toronto/Rio plasmids, TEM-220 (M182T and A185T substitutions) described in the Toronto/Rio plasmids, TEM (E110K substitution) described in the Africa plasmid, TEM (G228S substitution) described in the Africa plasmid and TEM (Q269K substitution) in penicillinase plasmids, TEM-75 (L21F, R164H and T265M substitutions; 2be group-ESBL), TEM-141 (K34E substitution; 2b group), TEM-198 (T271I substitution) [67, 70–73]. In addition, mutations causing P14T and P14S amino acid substitutions in the TEM-1 leader peptide, occurring in the Africa and Asia plasmids, have been described, which may be important in the expression of *bla* genes [71].

Practically, all TEM beta-lactamases described so far in *N. gonorrhoeae* have a narrow substrate spectrum that comprises penicillins, characteristic of group 2b beta-lactamases according to Bush classification [74, 75]. TEM-75 the only ESBL is very rare, and *N*. *gonorrhoeae* strain containing the plasmid was susceptible to ceftriaxone. The expression of the plasmid *bla* gene is probably dependent on two factors located in the chromosome: an inducer and a negative regulator of beta-lactamase expression. One may express a concern that the massive use of ESC in many countries for gonorrhoea treatment creates possibility of selecting new variants of TEM beta-lactamase, among which extended spectrum beta-lactamases (ESBLs) belonging to the 2b group, hydrolyzing oxyimino-beta-lactams, including ceftriaxone and cefixime, seem particularly dangerous. This appears particularly likely for a large number of beta-lactamase-producing strains. Although the number of *N. gonorrhoeae* strains producing beta-lactamases is not high in most European countries, the percentage of such strains in some Asian countries, such as Thailand, India and Bhutan is very high and in recent years ranged from 84 to 89% [76–78].

The highest percentage of *N. gonorrhoeae* producing beta-lactamase in Europe in 2016 was recorded in Sweden (24%), Austria (23.1%), Czech Republic (21.1%), Greece (20%), Malta (20%), Poland (18%) Slovakia (17,3%), Norway (16,2%), Belgium (16,2%) and Spain (16.2%). In Poland, we observed an increase in the number of beta-lactamase-producing *N. gonorrhoeae* strains from 1.1% in 2006 to 18% in 2016 [62, 63].

## Resistance to macrolides

Azithromycin is one of the most commonly used antibiotics from the group of macrolides, also in the treatment of sexually transmitted diseases. The mechanism of azithromycin action is based on the inhibition of bacterial protein synthesis as a result of binding to the V domain of the 23S rRNA, within the 50S subunit of the bacterial ribosome.

*N. gonorrhoeae* azithromycin resistance can have many causes. Typically, the MIC values for azithromycin are determined by several mechanisms occurring simultaneously. Probably, mutations in the gene encoding the 23S rRNA V domain are the most common cause of resistance to azithromycin in the currently dominant clones. Mutations can occur in all four gene alleles or in a fewer number of alleles. Two such mutations have been described [79]. The C2611T mutation, whose presence in three or four alleles of the gene, determines the azithromycin MIC at the level of 2–16 mg/L, and the presence of mutations in only one allele determines MIC in the range of 0.06–0.125 mg/L, i.e., such as in strains without mutations. The C2611T mutation, together with the overproduction of MtrCDE pump proteins, is likely the cause of resistance to azithromycin in majority of isolates belonging to the most widespread *N. gonorrhoeae* clone, NG-MAST ST1407, in Europe. The second mutation, A2059T, if it occurs simultaneously in three or four alleles of the gene, determines the high azithromycin MIC values of > 256 mg/L, and the presence of mutations in only one allele does not increase the MIC value [80, 81]. The third type of mutation, A2143G, occurring in all four alleles and conditioning azithromycin MIC of > 256 mg/L, has been described in some strains isolated in England [82].

The second group of mechanisms reducing the susceptibility to macrolides occurring in *N. gonorrhoeae* is the overproduction of MtrCDE or MacAB membrane pump proteins [83, 84]. The MtrCDE pump removes beta-lactams, macrolides, tetracyclines, rifampicin and detergents. It does not cause resistance alone, but increases the MIC value. The operon encoding the MtrCDE membrane pump is regulated by the MtrR repressor and the MtrA activator. The MtrR protein is involved not only in the regulation of the MtrCDE pump, but also in the regulation of approximately 65 other genes [85]. Overproduction of pump proteins may be caused by mutations in the promoter region of the *mtrR* gene (e.g., deletion of adenine at the 35 bp position preceding the *mtrR* gene), mutations within the *mtrR* gene and mutations conditioning the formation of a new *mtrCDE* promoter, the so-called *mtr120* not regulated by MtrR and MtrA [83, 85–87]. These mechanisms, occurring alone, are able to increase azithromycin MIC to 0.5 mg/L, and thus are not able to condition azithromycin resistance. When combined with other resistance mechanisms, they increase the MIC values. The MacAB pump removes only macrolides, and its overproduction is related to *macAB* operon promoter mutations [84, 87].

The third group of mechanisms is the synthesis of 23S rRNA methylases: ErmB, ErmF, ErmC, ErmA (they cause dimethylation of adenine A2058 within domain V of 23S rRNA) and synthesis of the transferable MefA/E membrane pump. These mechanisms are characteristic of many Gram-positive bacteria [88]. Genes conditioning these mechanisms are present in plasmids or transposons and were uptaken by *N. gonorrhoeae* from other bacteria. These mechanisms occurred in *N. gonorrhoeae* with a high frequency in strains isolated in 1940–1987 [28, 86]. They are very rare or non-existent in the currently isolated strains [24].

Highest percentage of *N. gonorrhoeae* resistant to azithromycin in 2016 (EUCAST breakpoint was MIC > 0.5 mg/L) occurred in Portugal (34.5%), Norway (16.2%), Hungary (16%), Greece (14%), Iceland (14.3%), Greece (39.6%), Ireland (37.6%), Latvia (14.3%) and France (10.9%) [61]. Overall, 75 azithromycin-resistant *N. gonorrhoeae* strains isolated in 17 European countries from 2009 to 2014 contained the 23S rRNAA2059G mutation in all four alleles (in four cases MIC ≥ 256 mg/L) or C2611T mutation in two to four alleles of the gene (remaining 71 cases) [89]. The frequency of azithromycin and ciprofloxacin resistance in several European countries in 2015–2017 is presented in Table 5 [53, 90–92]. Another problem is that azithromycin can be less effective in oropharyngeal than urogenital gonococcal infections [93].

**Table 5:** Antimicrobial resistance of *Neisseria gonorrhoeae* in Europe

Country	% of resistance*	
	Year: 2014/2015/2016/2017	
	Azithromycin	Ciprofloxacin
Austria	2.0/3.3/4.7/3.8	52.5/65.6/65.6/50.0
Belgium	3.6/3.0/9.1/12.2	57.9/49.5/44.4/46.9
Denmark	3.7/2.7/1.8/12.7	33.0/30.9/18.9/28.0
France	10.9/5.7/7.1/6.4	50.9/41.9/37.4/34.5
Germany	2.8/1.8/0.9/4.5	63.2/61.5/58.7/61.0
Greece	39.6/22.0/14.0/7.9	70.0/77.0/60.0/55.1
Hungary	1.1/4.7/16.0/8.1	55.1/53.1/40.4/46.8
Iceland	0.0/0.0/14.3/11.6	58.3/28.6/77.1/42.5
Ireland	37.6/18.2/15.0/9.8	34.7/45.5/42.0/51.5
Italy	6.0/2.0/11.0/16.0	78.0/71.0/53.0/58.0
Malta	4.8/13.8/8.0/25.9	57.1/65.5/44.0/63.0
Netherlands	1.8/4.0/2.0/5.6	32.2/37.0/29.4/31.0
Norway	5.5/3.6/16.2/14.0	73.6/58.7/46.0/43.9
Poland	8.7/5.4/2.6/12.3	65.2/57.1/57.1/76.9
Portugal	17.3/17.3/34.5/12.7	36.4/37.3/46.4/46.4
Slovakia	3.7/1.9/0.9/6.4	67.9/53.8/56.4/62.7
Slovenia	2.4/0.0/8.5/2.3	45.1/34.9/33.0/42.1
Spain	6.6/3.0/4.1/5.5	67.5/65.3/57.5/53.9
Sweden	4.0/14.0/5.0/3.0	57.0/45.0/47.0/35.0
United Kingdom	0.9/12.6/3.0/11.3	33.3/39.7/29.6/34.6
EU/EEA	7.9/7.1/7.5/7.5	50.7/49.4/46.5/46.5

*EU/EEA* European Union/European Economic Area

*Azithromycin, breakpoint MIC > 0.5 mg/L (EUCAST 2014–2017; ciprofloxacin, breakpoint MIC > 0.06 mg/L (EUCAST 2014–2017)

## Resistance to fluoroquinolones

The mechanism of ciprofloxacin action is based on the inactivation of two bacterial enzymes: topoisomerase II (gyrase) and topoisomerase IV, responsible for DNA superspiralization. *N.gonorrhoeae* ciprofloxacin resistance is most often conditioned by mutations in the *gyrA* and *parC* genes, coding for topoisomerases II and IV, respectively. Simultaneous combination of changes in both proteins usually determines the resistance. There are several most frequently described substitutions, but they occur at different frequencies depending on the geographical region. The most frequently described substitutions in the *N. gonorrhoeae* GyrA protein include: S91F or T [94–96], A92P, D95N or A or G, I97M and Q102H [94, 96]. The most frequently described substitutions in the *N. gonorrhoeae* ParC protein include: D86N, S87N or I or R, S88P, E91K or G or A and L106I. Synonymous (silent) mutations that did not change the amino acid were also described in the *parC* gene, e.g., codons Y104 (TAT/TAC), A129 (GCG/GCA), L131 (CTC/CTG) [94, 96]. *N. gonorrhoeae* strains that are highly resistant to fluoroquinolones usually have a combination of three or four mutations. The most common combinations of amino acid substitutions in the GyrA and ParC proteins conditioning resistance to fluoroquinolones are: S91F +D95G/A in GyrA and S87R in ParC. This combination was found in more than 40% of *N. gonorrhoeae* strains resistant to fluoroquinolones and conditioned ciprofloxacin MIC from 4 to 32 mg/L [96]. Other common combinations include: S91F + D95A in GyrA and D86N in ParC (11% of strains, ciprofloxacin MIC from 4 to 32 mg/L) and S91F + D95N in GyrA and S87N in ParC (10% of strains, ciprofloxacin MIC from 1 to 2 mg/L) [96]. The frequency of individual mutations in the *gyrA* and *parC* genes is varied. Mutations conditioning changes in the GyrA protein in the group of strains isolated in India, in 2007–2009: S91F or T, D95G/N, occurred in all strains, and mutation conditioning the change in the ParC protein: E91G in 46.9% of strains [95]. Mutations conditioning changes in the GyrA protein in the group of strains isolated in Brazil, in 2006–2010 occurred at the following frequencies: S91F (40%), D95G (40%), Q102H (12%), D95Y (4%) and mutations conditioning changes in the ParC protein occurred at a frequency of: S87R (40%), S88T (4%) [97]. S91F and D95G substitutions of amino acids in the GyrA protein and the S87R substitution in the ParC protein were reported in the most common European clone, ST1407, in ciprofloxacin-resistant strains (MIC 16–32 mg/L) [24]. All *N. gonorrhoeae* strains isolated in England and Wales in 2005–2009 showed high ciprofloxacin resistance (MIC ≥ 16 mg/L), 74% was the ST1407 clone [98].

A mechanism increasing fluoroquinolone MIC values, based on overproduction of NorM membrane pump proteins, was also described in single *N. gonorrhoeae* strains [95–97, 99]. The highest percentage of ciprofloxacin-resistant *N. gonorrhoeae* among European countries in 2016 was recorded in Iceland (77.1%), Croatia (66.7%), Austria (65.6%), Germany (58.7%) [61].

## Resistance to tetracyclines

Tetracyclines are not currently used in Europe for the treatment of *N. gonorrhoeae* infections as monotherapy, which is due to significant resistance. However, both tetracycline and doxycycline are sometimes used in a combination therapy, with third-generation cephalosporins, alternatively to azithromycin, mainly in mixed infections, e.g., with gonorrhoea and chlamydia coexistence [100–104].

The mechanism of antibacterial activity of tetracyclines results from their ability to bind to the 30S ribosomal subunit, which leads to protein synthesis inhibition. Resistance in most bacteria is associated with the presence of one of the two basic mechanisms. The first is based on removing the antibiotic from the cell by a variety of membrane pumps, e.g., proteins from TetA to TetL, belonging to the MFS superfamily. The second results from the active protection of the target site on the ribosome 30S subunit by proteins named from TetM to TetW [88, 105]. Resistance to tetracyclines in *N. gonorrhoeae* may be conditioned by the conjugative plasmids encoding the TetM protein (active ribosome protection) and then tetracycline MIC is 16–64 mg/L. It was found at the beginning of the 1990s that conjugative plasmids, conditioning tetracycline resistance in *N. gonorrhoeae*, occur in two types, both 25.2 MDa and both contain the *tetM* gene. Differences were demonstrated on the basis of restriction mapping and southern blot hybridization. One type was named Dutch and the other type American [106, 107]. Sequencing of the *tetM* genes conducted in later years also showed the presence of two types, which were named Dutch and American types, respectively, taking the name from the source plasmid type. It is currently known that the plasmid type is usually, but not always, compatible with the type of the *tetM* determinant [108]. Other mechanisms resulting from mutations in chromosomal genes are responsible for tetracycline resistance in epidemic strains, e.g., *penB* encoding the B porin protein [25, 109], *penC* encoding PilQ secretin [110], *rpsJ* encoding a ribosomal S10 protein [94, 111] and overproduction of MtrCDE membrane pump proteins, associated with mutations in promoter regions of *mtrR* and *mtrC* genes and the *mtrR* gene [83, 109, 112]. These mechanisms condition a significantly lower level of resistance, which determines the tetracycline MIC at 2–4 mg/L.

It was demonstrated that the Dutch-type plasmids were more frequent in Asian countries than American ones among *N. gonorrhoeae* strains showing a high level of resistance to tetracyclines, e.g., they represented over 99% plasmids in China, similarly in Indonesia, Philippines, Thailand [57, 113, 114] and Bangladesh [115]. The Dutch type was also predominant in the countries of South America, such as Brazil, Guyana or Trinidad [116, 117].

Studies conducted in some European countries, involving strains resistant to tetracyclines, isolated in 1988–1995, showed that American-type plasmids were more common (81.8%) than the Dutch ones [114]. The Dutch type was dominant among strains isolated in our center in Warsaw in 2013 (88.9%) [118].

The incidence of tetracycline resistance in *N. gonorrhoeae* depends on the period and country where bacteria were isolated. In China, the incidence of *N. gonorrhoeae* strains with the *TetM* determinant increased 18-fold in 1999–2005, reaching over 32% [113]. The frequency of tetracycline resistance in Poland in 2010–2013 was found to remain at a similarly high level (from 40 to over 50%) during this period [63, 118]. Similarly, a high percentage of *N. gonorrhoeae* strains resistant to tetracycline was recorded in Germany (over 41%) [119], Belarus (40%) [120] and Indonesia, where the percentage of resistant strains was even 100% in 2010–2012 [76]. Fewer, but also a significant number of tetracycline-resistant strains were found in a similar period in India (12%) [77], Sri Lanka (16.3%) [76] and Russia (16.9%) [94].

## Resistance to spectinomycin

Spectinomycin belongs to the aminocyclitol antibiotics and, like other drugs in this group, binds to the bacterial 30S ribosomal subunit, which results in protein synthesis inhibition. Spectinomycin has been used since the early 1960s, basically exclusively for gonorrhoea treatment. The application of spectinomycin was limited to penicillin-allergic patients or in case of therapeutic failure with penicillin therapy, most often resulting from the production of beta-lactamase by *N. gonorrhoeae* strain. Although *N. gonorrhoeae* strains with a high level of resistance (MIC = 2048 mg/L) [121, 122] were already described in the late 1970s, current studies demonstrate that this resistance is extremely rare or virtually non-existent. Despite the high susceptibility of *N. gonorrhoeae* strains, spectinomycin is not recommended as a first-line drug for gonorrhoea treatment in Europe, because it is less effective in oropharyngeal infections due to poor distribution in the human body [123, 124]. The drug is not only ineffective in other sexually transmitted infections but also could delay incubation period and hinder diagnosis of syphilis. There is also a problem with spectinomycin accessibility in many European countries and in North America. However, due to the low price and considerable effectiveness, it is still used as an alternative medicine in some Asian countries, such as South Korea or in South America [84, 125].

The reported resistance to spectinomycin in *N. gonorrhoeae* is mutational, and mutations affect either 16S rRNA-encoding genes, specifically *rrs*16S rRNA (G1064C, C1192U or C1192T) [126] or the *rpsE* gene encoding the S5 ribosomal protein [127]. Resistant strains, as opposed to, e.g., penicillin-resistant ones, have not spread and are very rare [128, 129]. For example, the frequency of resistant strains in a study of over 4500 isolates from six Asian countries was found to be 0.17% [60]. Most often, this type of research does not detect resistant strains [4, 24, 120].

## Conclusion

In Europe, gonorrhoea is usually treated with either ceftriaxone monotherapy or, more preferably, ceftriaxone in combination with azithromycin. The recent emergence of *N. gonorrhoeae* strains resistant to ceftriaxone and the threat of their spread and the gradual increase in a proportion of azithromycin-resistant strains may in the future lead to major difficulties in empiric treatment of this disease. There is a risk of spreading of existing ceftriaxone- and cefixime-resistant clones, especially the resistant variants of the clonal CC1 complex (G1407 genogroup). Evolution of TEM beta-lactamases to ESBL is also possible, similarly as in Gram-negative bacilli. However, so far, cefixime resistance among *N. gonorrhoeae* isolated in Europe in 2009–2017 has remained at around 2% with no apparent upward trend. In addition, a high percentage of resistance to ciprofloxacin (46.5%) and azithromycin (7.5%) was found in *N. gonorrhoeae* isolated in Europe in 2017. Introduction of new drugs is possible in the perspective of the next few years. On the other hand, returning to spectinomycin treatment, not used in Europe, seems unlikely due to numerous limitations, especially in the treatment of infections in oropharyngeal location.
